# 8-Oxoguanine: from oxidative damage to epigenetic and epitranscriptional modification

**DOI:** 10.1038/s12276-022-00822-z

**Published:** 2022-10-21

**Authors:** Ja Young Hahm, Jongyeun Park, Eun-Sook Jang, Sung Wook Chi

**Affiliations:** 1 Department of Life Sciences, Korea University, Seoul, 02481 Republic of Korea; 2 Institute of Life Sciences and Biotechnology, Korea University, Seoul, 02481 Republic of Korea; 3 KU-KIST Graduate School of Converging Science and Technology, Korea University, Seoul, 02481 Republic of Korea

**Keywords:** RNA modification, DNA adducts, DNA metabolism

## Abstract

In pathophysiology, reactive oxygen species control diverse cellular phenotypes by oxidizing biomolecules. Among these, the guanine base in nucleic acids is the most vulnerable to producing 8-oxoguanine, which can pair with adenine. Because of this feature, 8-oxoguanine in DNA (8-oxo-dG) induces a G > T (C > A) mutation in cancers, which can be deleterious and thus actively repaired by DNA repair pathways. 8-Oxoguanine in RNA (o^8^G) causes problems in aberrant quality and translational fidelity, thereby it is subjected to the RNA decay pathway. In addition to oxidative damage, 8-oxo-dG serves as an epigenetic modification that affects transcriptional regulatory elements and other epigenetic modifications. With the ability of o^8^G•A in base pairing, o^8^G alters structural and functional RNA–RNA interactions, enabling redirection of posttranscriptional regulation. Here, we address the production, regulation, and function of 8-oxo-dG and o^8^G under oxidative stress. Primarily, we focus on the epigenetic and epitranscriptional roles of 8-oxoguanine, which highlights the significance of oxidative modification in redox-mediated control of gene expression.

## Genetics: modification of guanine base has far-reaching consequences

Emerging evidence suggests the modified base 8-oxoguanine, created when damaging chemicals called reactive oxygen species interact with DNA and RNA, has more extensive effects on cell function than previously suspected. This modified form of guanine, one of the key chemical ‘bases’ of DNA and RNA can cause mutations in DNA and disrupt the functioning of the RNA copies of DNA that mediate gene expression. These problems occur because 8-oxoguanine can aberrantly pair up with the base adenine in crucial DNA and RNA interactions, generating mutations that can cause cancers and other conditions. Sung Wook Chi and colleagues at Korea University in Seoul, review research offering evidence of the wider consequences of 8-oxoguanine formation, especially disruption of the complex processes that regulate the activity of genes and RNA. Further investigation of the medical implications is warranted.

## Introduction

Reactive oxygen species (ROS), including hydroxyl radicals, superoxide, and hydrogen peroxide (H_2_O_2_), are continuously generated as byproducts of aerobic metabolism (e.g., cellular respiration in the mitochondria)^1,2^. The ROS concentration must be balanced to maintain a normal redox state and hence actively controlled by antioxidant pathways. However, increasing ROS production induced by environmental stress or pathophysiological conditions overwhelms homeostatic regulation, thereby imposing oxidative stress. Oxidative stress is involved in various pathogeneses, including tumorigenesis and neurodegenerative disorders^1,2^. Depending on the concentration and compartmentalization of ROS, oxidative stress differentially oxidizes biomolecules such as lipids, proteins, and nucleic acids, resulting in varying effects on redox signaling as second messengers or on cellular components as oxidative damage^2^.

**Fig. 1:**
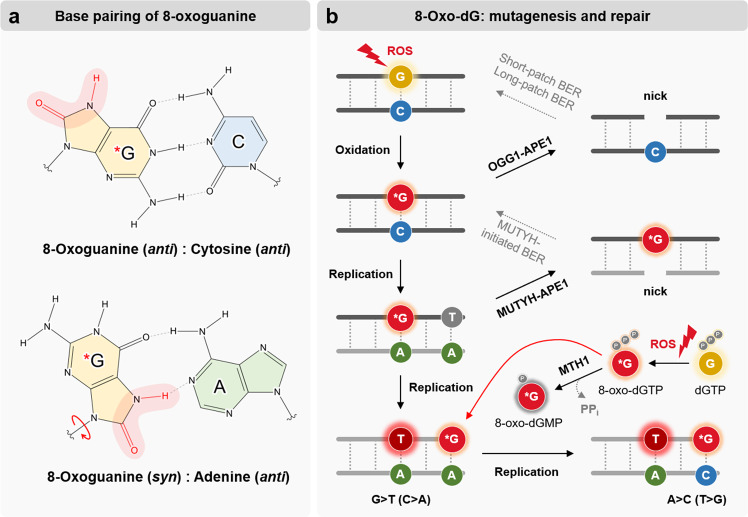
Characteristics of 8-oxoguanine underlying base pairing, mutagenesis, and DNA repair. **a** 8-Oxoguanine (*G), formed by reacting with oxygen at the C8 position (highlighted in red), pairs with cytosine (C) through its anticonformation (*G•C; upper panel). 8-Oxoguanine (*G) in syn conformation uses a Hoogsteen edge to pair with adenine (*G•A; lower panel). **b** 8-Oxo-dG-induced mutagenesis and its repair pathways. 8-Oxo-dG (*G) is recognized and removed by OGG1, subsequently processed into nicks by APE1 and repaired either by short-patch or long-patch BER (upper panel). *G•A mismatch is recognized by MUTYH, followed by the APE1 reaction, and repaired by MUTYH-initiated BER (middle panel). Unrepaired 8-oxo-dG results in G > T transversion (same as C > A) during DNA replication (lower left panel). ROS induce oxidation of free nucleotide (8-oxo-dGTP), which is actively hydrolyzed by MTH1 (8-oxo-dGMP) to prevent its incorporation from DNA replication. The unrepaired 8-oxo-dG results in an A > C mutation (same as T > G; lower panel).

Among the oxidative modifications, the guanine of nucleic acids susceptibly forms 8-oxoguanine (8-oxo-7,8-dihydroguanine), a tautomer known as 8-hydroxyguanine^3^. 8-Oxoguanine was first discovered in DNA during the characterization of carcinogenic molecules related to oxidative stress^4^; thus, it has been widely used as a ROS biomarker^1,5^. 8-Oxoguanine can be either produced directly at the DNA (8-oxo-dG) and RNA (o^8^G) levels or at the free nucleotide level (8-oxo-dGTP or o^8^GTP), which can be incorporated through DNA replication^6^ or RNA transcription^7^. The critical feature of 8-oxoguanine is that its *syn* conformation uses a Hoogsteen edge to base pair with adenine, whereas its *anti* conformation still pairs with cytosine as an unoxidized guanine^6^ (Fig. 1a). Therefore, 8-oxo-dG causes guanine-to-thymine transversion, causing mutations (G > T, the same as C > A)^8^, especially in the cancer genome^9^. To prevent this damage, 8-oxoguanine DNA glycosylase (OGG1) recognizes, removes, and repairs 8-oxo-dG via base excision repair (BER) pathways^10^ (Fig. 1b). In addition to changes in genetic information, 8-oxo-dG, particularly produced through physiological metabolism, acts as an epigenetic marker that affects regulatory elements in promoters, methylation of CpG islands, and distribution of histone modifications, thereby regulating gene expression.

Upon exposure to oxidative stress, guanine in RNA is more vulnerable to producing o^8^G than guanine in DNA, but less attention has been given to o^8^G because RNA is a relatively unstable and temporal intermediate^11^. Nonetheless, inappropriate o^8^G•A base pairing impacts RNA structure and functions at the posttranscriptional level; o^8^G induces translational errors, and its extensive occurrence in mRNA deteriorates translational activity^11,12^. Therefore, damaged RNAs generally undergo decay via surveillance mechanisms for RNA quality control^12^. Beyond damage, o^8^G can serve as an epitranscriptional modification that alters regulatory RNA–RNA interactions in a redox-dependent manner^12,13^.

Currently, there are an increasing number of studies on the epigenetic^14,15^ and epitranscriptional^16^ roles of 8-oxoguanine. Here, we review the functions of 8-oxoguanine as an oxidative modification in DNA (8-oxo-dG) and RNA (o^8^G), describing oxidative damage, which necessitates repair or quality control, and regulatory mechanisms for redox-mediated gene expression at the transcriptional and posttranscriptional levels.

## 8-Oxoguanine in DNA

### 8-Oxo-dG

As accurate transmission of genomic information is essential to preserve genome stability, DNA must be protected from the accumulation of mutations. Genomes are continually threatened by chemical assaults, including ROS, alkylating reagents, ultraviolet light, and carcinogens^17^. It is estimated that an average of ~70,000 nucleobases are damaged in each human cell every day^18^. As damaged DNA can result in deleterious mutations, cells trigger specific DNA damage responses to defend and repair these changes (e.g., cell cycle checkpoint and BER pathways)^17^. Among this damage, DNA oxidation is the most abundant and relevant to diverse redox-mediated biological consequences (e.g., inflammation and stress-induced premature senescence)^14^. Currently, over 100 oxidative DNA adducts have been identified, ranging from those with modifications of the bases (e.g., 8-oxo-dG, 8-oxo-dA, thymidine glycol, 5-hydroxylcytosine, and 5-hydroxyuracil) and nucleotides (abasic or cyclic forms; e.g., 2-deoxyribonolactone, 5′,8-cyclo-2’-deoxyguanosine, and 5′,8-cyclo-2’-deoxyadenosine) to those with breakage of the phosphate backbone^15^.

Due to the lowest redox potential of guanine relative to the other bases (G: –3.0 V, A: –2.71 V, C: –2.56 V, and T: –2.32 V)^19^, 8-oxo-dG is the most prevalent oxidized form generated by reacting with oxygen at the C8 position, of which the double bond in guanine is directly attacked by the hydroxyl radical (^•^OH). 8-Oxo-dG is estimated to be present at approximately 0.5 per Mbp (millions of base pairs; steady state of the human lymphocyte genome)^20^. The redox potential of guanine oxidation is largely affected by the flanking sequence composition, at which purine-rich sequences, specifically guanine at the 5′-end or GG repeats, and those neighboring oxidized bases are favored, presumably due to the migration of radical cations^21,22^. Two or more oxidative lesions often occur within 10 bp, called oxidative clustered DNA lesions (OCDLs)^23^. The OCDL level is 0.02 to 0.8 per Mbp in normal human primary and cancer cells^24,25^. Some OCDLs seem more difficult to repair than individual lesions^23,26^ and are thus more likely to induce pathological mutations^23,26^.

8-Oxo-dG is highly mutagenic because of its propensity to pair with adenine in a *syn* conformation (Fig. 1a), causing a guanine-to-thymine mutation (G > T, the same as C > A) during DNA replication^6,8^. DNA polymerase β (pol β) accommodates the 8-oxo-dG template in the *syn* conformation, hence incorporating adenine into the replicating strand (Fig. 1b). 8-Oxo-dG can be formed not only in DNA molecules but also in free nucleotides (Fig. 1b), the pools of which are especially vulnerable to oxidative damage (8-oxo-dGTP)^27^. As 8-oxo-dGTP provokes changes in the active site of pol β, its *syn* conformation can be inserted in the opposite adenine, avoiding recognition as damaged, thus resulting in an A > C mutation (the same as T > G) termed polymerase-induced cytotoxicity (Fig. 1b)^28^.

### 8-Oxo-dG repair pathways

BER is a DNA repair mechanism that corrects small base lesions unless the DNA helix is distorted^29^. First, the damaged base is removed by DNA glycosylases, which have broad substrate specificity to aid in fast repair^30^. The remaining apurinic/apyrimidinic (AP) site is further processed through endonuclease activity, and the AP sugar-phosphate backbone is cleaved to form a single-strand break (SSB). Then, the resulting gap in the SSB is filled and rejoined by replacing the AP site with a proper single-nucleotide match (short-patch BER) or by synthesizing a few long matches (a stretch of 2–10 nucleotides, long-patch BER) to correct the damage^31^. A wide variety of glycosylases are used in BER to repair different types of damage, such as those induced by oxidation (e.g., 8-oxo-dG, 8-oxo-dA, and formamidopyrimidine, such as fapyG or fapyA), alkylation (e.g., 3-methyladenine and 7-methylguanosine), and deamination (e.g., hypoxanthine, xanthine, and uracil)^32^. Examples of DNA glycosylases for damaged bases include OGG1 for 8-oxoguanine, Mag1 for 3-methyladenine, and UNG for uracil. Depending on their AP lyase activity, DNA glycosylases are divided into two classes, monofunctional and bifunctional. Separate AP endonucleases (APE1 and APE2) are required for monofunctional DNA glycosylases, whereas bifunctional endonucleases are sufficient to produce SSB for BER^33^.

OGG1 is primarily responsible for removing 8-oxo-dG (Fig. 1b); it excises 8-oxo-dG opposite to the cytosine base and generates an AP site^10^. OGG1 is a bifunctional DNA glycosylase capable of cleaving the 3′-end of the AP site; 3′-deoxyribose phosphate (3′-dRP) and 5′-phosphate are produced via a *β*-elimination mechanism. In addition, apurinic/apyrimidic endonuclease 1 (APE1) catalyzes the hydrolysis of the phosphodiester bond at the 5′-end of the AP site, yielding 3′-hydroxyl (OH) and 5′-dRP at the termini^32^. Although both enzymes cleave the backbone at the AP site, because of the low efficiency of the AP lyase in OGG1, they produce the AP site or single-nucleotide gap, harboring different types of unconventional DNA ends (AP endonuclease, 3′-OH, 5′-dRP; AP lyase, 3′-dRP, 5′-phosphate)^15^. The produced SSBs are detected and occupied by poly(ADP-ribose) polymerase 1 (PARP1) and PARP2, which synthesize poly(ADP-ribose) (PAR) and activate PARylation at the damaged site, resulting in the rapid recruitment of downstream repair proteins (e.g., pol β and X-ray repair cross-complementing protein 1, XRCC1) and relaxation of the chromatin structure^29^. PAR synthesis from PARP1 is also involved in 8-oxo-dG BER, which is reported to be mediated by the nuclear membrane protein lamin A^34^.

The dRP lyase activity of pol β is used to fill and ligate SSB, and the process undergoes either short- or long-patch BER. In short-patch BER, pol β excises downstream of 5-dRP and inserts a single nucleotide into the gap. Then, the nick in the incorporated site is ligated by DNA ligase III and complexed with XRCC1^35^. In long-patch BER, which is frequently used for OCDL, pol β inserts the first nucleotide, and the remaining nucleotides are subsequently elongated by other replicative DNA polymerases (pol δ and/or ε)^36^. The “flap” structure produced is resolved by flap endonuclease 1 (FEN1), which removes displaced oligonucleotides and is sealed by DNA ligase I. Additionally, several accessory proteins are required for a successful repair. Proliferating cell nuclear antigen (PCNA) helps pol δ to properly synthesize a repaired strand by serving as a DNA sliding clamp and interacts with FEN1 to stimulate its excision activity^37^. Replication factor C (RFC) facilitates PCNA loading^38^^,^ and replication protein A (RPA) stabilizes the newly synthesized DNA strand for pol δ or pol ε^39^.

8-Oxo-dG can be repaired by MutY DNA glycosylases (MUTYHs; Fig. 1b), which remove bases including adenine when inappropriately paired with 8-oxo-dG^40,41^. Since MUTYH is monofunctional, the AP site opposite of 8-oxo-dG is only excised by APE1, replaced with cytosine-containing nucleotides by DNA polymerase λ (pol λ) as a complex with PCNA and RPA, processed by FEN1, and ligated by DNA ligase I^29^. Additionally, 8-oxo-dG is removed by Nei-like DNA glycosylase 1 (NEIL1), homologous to bacterial fapy-DNA glycosylase (Fpg), which removes diverse oxidized bases, including 8-oxo-dG, but NEIL1 mainly functions in oxidized pyrimidines and ring-opened purines (e.g., fapyG and fapyA)^36^.

In addition to BER, other repair systems can be used to treat 8-oxo-dG. As a fundamental mechanism for the clearance of 8-oxo-dG from the nucleotide pool, MutT homolog 1 (MTH1) hydrolyzes 8-oxo-dGTP in cells to prevent DNA polymerase from incorporating it^42^ (Fig. 1b). In addition, transcription-coupled nucleotide excision repair (TC-NER) can also remove 8-oxo-dG in the transcribed strand, where Cockayne syndrome B (CSB)^43^ and xeroderma pigmentosum complementation group C (XPC)^44^ recruit and activate APE1 for removal of the oxidized lesion. After DNA replication, the remaining 8-oxo-dG mismatch (8-oxo-dG•A) has the opportunity to be removed by mismatch repair (MMR), in which MMR proteins (e.g., MSH2/6) play a role with MUTYH and PCNA^41^. Moreover, other enzymes, such as N-methylpurine DNA glycosylase (MPG)^45^ and 40 S ribosomal protein S3 (RPS3)^46^, have the capacity to cleave 8-oxo-dG-containing DNA. As summarized in Table 1, 8-oxo-dG repair pathways have overlapping substrate specificities and recognition, serving as backups for the main repair pathway mediated by OGG1 in BER.

**Table 1:** 8-oxo-dG repair pathways.

8-Oxo-dG repair pathway	DNA lesion (substrate)	Damage recognition and removal	Downstream repair proteins	Reference
Base excision repair (BER)	8-oxo-dG	OGG1 → APE1	Short-patch BER: DNA pol β, DNA ligase III/XRCC1	^10,31,35^
		OGG1 → APE1	Long-patch BER: DNA pol β, DNA ligase I, FEN1, PCNA, RFC, RPA, DNA pol δ or pol ε	^29,36–39^
	8-oxo-dG coupled with A	MUTYH → APE1	DNA pol β, DNA pol λ	^40^
	8-oxo-dG but mainly fapyG, fapyA	NEIL1 → PNK	DNA pol β, DNA ligase III/XRCC1	^36,42^
Hydrolysis	8-oxo-dGTP	MTH1	N/A	^42^
Transcription-coupled nucleotide excision repair (TC-NER)	8-oxo-dG	CSB, XPC → APE1		^43,44^
Mismatch repair (MMR)	8-oxo-dG coupled with A	MSH2/6, MUTYH	PCNA	^41^
Others	8-oxo-dG	MPG, RPS3	Unknown	^45,46^

### 8-Oxo-dG-induced mutation and genome instability in cancer

8-Oxo-dG is involved in the pathogenicity of ROS-related diseases such as premature aging, neurodegeneration, and cancer^9,14,47^. Although chromosomes are continuously monitored and repaired by DNA repair enzymes, oxidative formation of 8-oxo-dG can easily accumulate by the overload of free radicals and induce harmful mutations, which are frequently observed in cancer with deficiency of a specific 8-oxo-dG repair mechanism. Impairment of 8-oxo-dG repair increases genomic alterations, particularly in cancer^47^. 8-Oxo-dG-induced G > T mutation (also C > A) was initially proven to be detrimental using the proto-oncogene HRas with synthetic 8-oxo-dG (codon 12: G^GGC^ > V^GTC^, codon 61: Q^CAG^ > K^AAG^)^48^. Mutagenic 8-oxo-dG•A pairing structurally mimics the T•A base pair, at which the repair proteins can hardly recognize the damage; however, promutagenic 8-oxo-dG•C base pairs can be readily recognized due to the different DNA helix structures^49^. Therefore, 8-oxo-dG should be repaired rapidly during this initial stage^29^.

In support of this observation, genetic studies have revealed the importance of 8-oxo-dG repair in cancer^47^. OGG1 knockout (KO) mice display elevated 8-oxo-dG concentrations, G > T mutation frequency, and susceptibility to genotoxic drug-induced tumor development, albeit displaying no other distinct phenotypic change. MUTYH KO^50^ and MTH1 KO^51^ spontaneously produce a higher tumor incidence, where an increased G > T frequency was more frequently observed in oxidative stress-induced tumorigenesis. Under KBrO_3_ treatment, MUTYH KO mice are prone to intestinal cancer^50,52^^,^ and MTH KO confers G > T mutations in the tumor suppressor APC gene, as observed in patients with tumors with MUTYH mutation^53,54^. OGG1/MUTYH double KO mice resulted in a G > T mutation in the KRAS (codon 12) oncogene and were prone to developing tumors (e.g., lung and ovarian cancers and lymphomas)^55^. Moreover, OGG1/MUTYH/MTH1 triple KO mice, which had a short lifespan and developed various types of tumors, displayed substantial accumulation of 8-oxo-dG causing spontaneous and inheritable de novo G > T mutations in the germline^56^.

Defects in 8-oxo-dG repair are often found in patients with cancer; for example, the OGG1 locus on chromosome 3p26.2 is frequently deleted in several cancer types^57^. The 8-oxo-dG-induced G > T mutation is widespread in cancer; copy number loss of OGG1 and MUTYH in patients with neuroblastoma causes high levels of G > T substitutions with a poor survival rate^58^. Sequencing analyses of coding regions in 518 protein kinase genes have revealed that G > T is a major somatic mutation in 210 diverse human cancers^59^. Mutation signatures with G > T are distinctly categorized as typical patterns in the analyses of single base substitutions (SBS) of the human cancer genome, SBS18 and SBS36 in the Catalogue of Somatic Mutations in Cancer (COSMIC) database^60^. SBS18 is proposed to be caused by ROS damage, commonly occurring in various cancer types as a background signature (e.g., neuroblastoma from unknown cause). SBS36 is a signature of defective BER caused by MUTYH mutations, often observed in endocrine pancreatic carcinoma and non-Hodgkin B-cell lymphomas^61^. Moreover, SBS signatures of tobacco smoking (SBS4) and tobacco chewing (SBS29) and defective DNA mismatch repair with microsatellite instability (SBS14) also confer a preference for the G > T mutation. In addition to cancer, embryonic cell cultures without antioxidants increase 8-oxo-dG levels, inducing mutations in the Tbx5i promoter and leading to cardiac-like differentiation^62^.

If DNA repair is not sufficient for fixing the present damage in the cell, the genome becomes unstable and promotes cell death or tumorigenesis^17^. Repair of 8-oxo-dG is important for maintaining genomic stability. Defects in OGG1 reduce cell viability^10^, and defects in MUTYH^52^, APE1^63^, and NEIL1^64^ induce the accumulation of SSBs caused by incomplete repair of 8-oxo-dG. Intriguingly, high BER activity impairs the maintenance of genome stability and leads to tumorigenesis^30^, presumably because it can accommodate overwhelming genomic mutations and instability rather than cell death. High levels of APE1 have been reported in many types of cancer, including prostate and ovarian cancers^65^. Moreover, incomplete or misrepaired 8-oxo-dG can hinder the delicate control of genome topology, resulting in genome destabilization^66^. Unrepaired 8-oxo-dG affects the catalytic activity of human topoisomerase I (TOPI), which plays a key role in DNA replication^67^. Transient cleavage by TOPI is indispensable for DNA replication to relax and unwind DNA without creating extreme torsional stress during the process. However, 8-oxo-dG increases the DNA-binding affinity of TOPI and promotes the overload of TOPI, inducing abnormal and excessive cleavage of DNA strands^66^. This increase in TOPI-DNA binding can potentially lead to DNA damage, cell death, recombination, and mutagenic effects. Finally, the 8-oxo-dG and AP sites can induce conformational changes in the DNA secondary structure. By stalling the DNA replication machinery, oxidized DNA can induce structural changes around the replication fork, interfering with DNA replication and causing genomic instability, thereby profoundly contributing to tumor development^68^.

### 8-Oxo-dG-induced transcriptional mutations in diseases

**Fig. 2:**
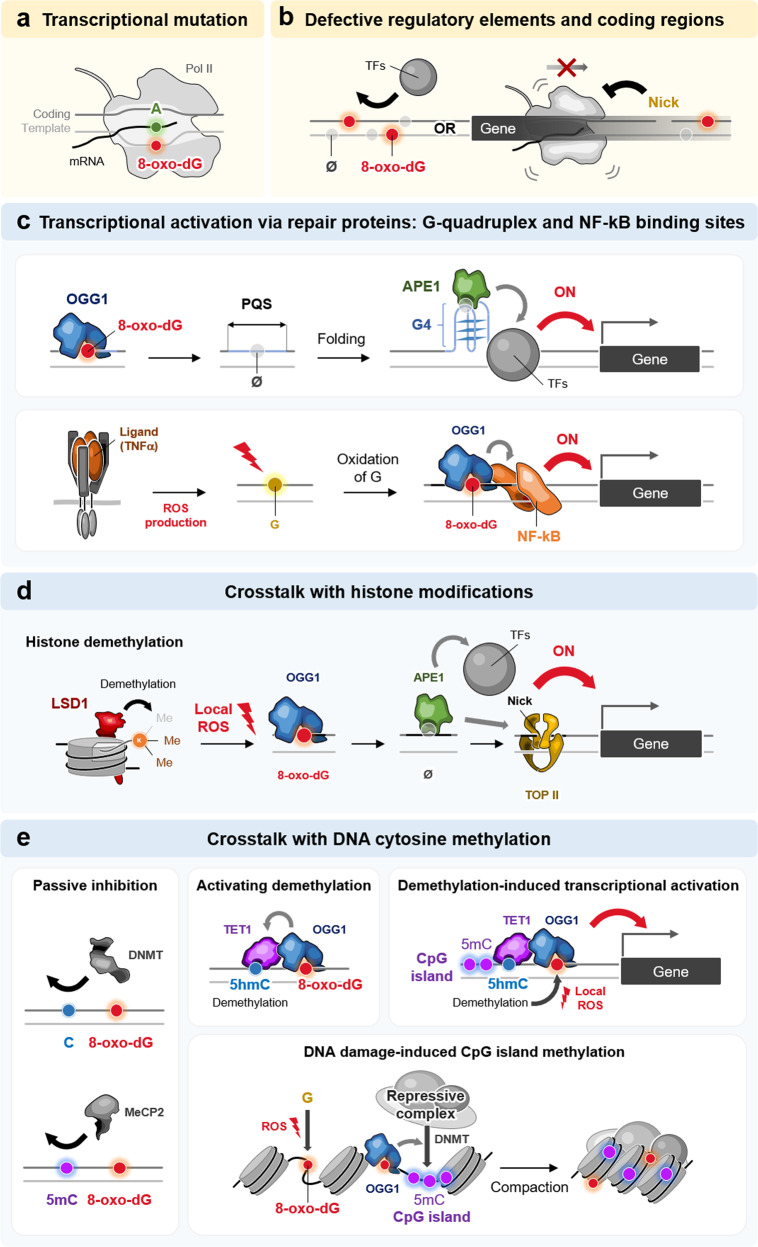
Epigenetic roles of 8-oxo-dG. **a** 8-Oxo-dG-induced transcriptional mutation, which is caused by 8-oxo-dG in the template strand during transcription, triggering a C > A point mutation in mRNA via 8-oxo-dG•A base pairing. **b** 8-Oxo-dG-induced loss of integrity in transcriptional regulatory elements. 8-Oxo-dG and its repair intermediate, AP site (Ø), deteriorate the integrity of transcriptional regulatory elements, thus hindering the binding of transcription factors (TFs). 8-Oxo-dG, AP sites, and subsequent nicks in coding regions also inhibit mRNA transcription. **c** Transcriptional regulation mediated by 8-oxo-dG and its repair proteins. 8-Oxo-dG, bound by OGG, and its intermediate AP site induce folding of quadruplex-forming sequences (PQS) into a G-quadruplex structure (G4), which recruits various TFs to transcriptionally activate downstream genes (upper panel). Ligand-mediated activation (e.g., TNFα) of the signaling pathway generates ROS, which oxidize DNA sequences near NF-kB binding sites. The produced 8-oxo-dG recruits the OGG1-NF-kB complex, thus activating the transcription of downstream genes (lower panel). **d** Interplay of 8-oxo-dG with epigenetic histone modifications. During the histone demethylation reaction, LSD1 generates local ROS (H_2_O_2_) that lead to the formation of 8-oxo-dG and AP sites in the promoter, which are occupied by OGG1 and APE1. Then, APE1 recruits other TFs, and its nick formation associates with topoisomerase II, eventually activating the transcription of downstream genes. **e** Interplay of 8-oxo-dG with DNA cytosine methylation (5mC). 8-Oxo-dG near CpG islands inhibits the binding of DNMT and MeCP2, thus passively interfering with 5mC (left panel). OGG1, which is associated with 8-oxo-dG, interacts with TET1, which oxidizes adjacent 5mC to 5-hydroxymethylcytosine (5hmC) for DNA demethylation (upper middle panel). During the DNA demethylation process of CpG islands, TET1 generates local ROS, which induce 8-oxo-dG associated with OGG1, thus activating the transcription of downstream genes (upper right panel). Oxidative DNA damage triggers the formation of the 8-oxo-dG and OGG1 complex, which recruits repressive complexes, including DNMT, and induces methylation of CpG islands, finally resulting in chromatin condensation and silencing of damaged DNA regions.

8-Oxo-dG modification not only alters DNA information during replication (G > T transversion) but also mediates mutations in transcription and regulates genetic information^14,15^. Despite the high fidelity of RNA polymerases, 8-oxo-dG in the template strand can be directly transcribed, resulting in C > A transversion in mRNA due to 8-oxo-dG•A base pairing. This phenomenon is called transcriptional mutation (TM)^69^, in which 8-oxo-dG located in the coding sequence leads to the translation of erroneous proteins (Fig. 2a), which are subjected to nonproliferating cells without undergoing DNA replication^70^. TM was initially demonstrated in *Escherichia coli*, in which 8-oxo-dG lesions produce mutant transcripts and defective activity of the luciferase reporter gene^71^. As 8-oxo-dG is bypassed by RNA polymerase II in vitro^72^, the luciferase reporter gene with 8-oxo-dG escapes transcription-coupled DNA repair and produces mutations in the transcripts and proteins, even in OGG1 KO mammalian cells^73,74^. TM is supported by a structural study showing that 8-oxo-dG can pair with adenine at the active site of RNA polymerase II, and the prerequisite of ATP incorporation appears to depend on base pairing at the adjacent upstream position^75^.

The physiological outcomes of TM have been implicated in tumor development and neuronal degeneration. 8-Oxo-dG in HRas was mutated in mRNA by TM and produced a constitutively active protein (codon 61: Q^CAG^ > K^AAG^), particularly under deficient BER (OGG1 KO) or transcription-coupled repair (CSB KO)^76^. As oxidative DNA damage correlates with functional impairment of nonproliferating neuronal cells, TM has been proposed as a mechanism to generate neurotoxic proteins, which may potentially cause α-SYN aggregation in Parkinson’s disease^70^. Moreover, 8-oxo-dG-induced TM can deteriorate splicing fidelity, in that the minigene splicing reporter of proteolipid protein 1 (PLP1), which harbors synthetic 8-oxo-dG, produces a detrimental DM20 splicing variant that causes X-linked leukodystrophy^77^.

### 8-oxo-dG-induced defective regulatory elements in aging

8-Oxo-dG and its repair intermediates (e.g., AP site) can affect gene expression by deteriorating the integrity of transcriptional elements (Fig. 2b). When the promoter regions of genes become oxidized, the activity of regulatory sequences, particularly those containing runs of guanine, becomes defective. Synthetic oligonucleotides, which contain 8-oxo-dG in transcription factor-binding sites, have reduced binding affinity for SP1^78^, NF-κB^79^, and CREB^80^. Given that the repair intermediates of 8-oxo-dG, processed by OGG1, are AP sites that contain no base for pairing, 8-oxo-dG is suggested to elicit significant repressive roles in transcription and by inducing transcriptional stalling in the coding regions. In support of this, 8-oxo-dG in the aged human brain with increasing ROS markedly accumulates in the promoter regions of transcriptionally decreasing genes (e.g., CaM1, Calb1, Calb2, sortilin, and PKCγ)^81^. Similarly, 8-oxo-dG and its repair intermediates, even those located in nontranscribed DNA strands of coding genes, also suppress transcription^82^, presumably by dysregulating regulatory elements in transcription, which usually function bidirectionally.

## Epigenetic roles of 8-oxoguanine

### Transcriptional regulation: G-quadruplex and NF-kB-binding site

8-Oxo-dG modification not only damages DNA information but also functions as an epigenetic marker that mediates transcriptional regulation together with its repair intermediates^14,15^. Although 8-oxo-dG changes and its repair intermediate, the AP site, loses base pairing information, such adducts can serve for the recruitment of repair proteins (e.g., OGG1 and APE1) to control transcription by actively interacting with other regulatory elements and structures^14,15^ (Fig. 2c). The synthetic modification of 8-oxo-dG in some promoter regions, possibly occurring in the VEGF^83^, TNFα^84^, BCL2^85^, and SIRT1^86^ genes, was initially found to activate the transcription of reporter genes. Intriguingly, although oxidative stress oxidizes the VEGF promoter, reduced binding of SP1 to G-rich elements increases transcription^87^. Later, the G-rich element was found to form a G-quadruplex structure (potential quadruplex-forming sequences; PQS), which can be thermodynamically driven by the AP site and processed from 8-oxo-dG by OGG1-mediated BER^88^. Furthermore, by recruiting APE1 to this AP site, the G-quadruplex structure enables the utilization of the redox-effector factor-1 (ref. ^1^) domain of APE1, independent of its catalytic activity, thereby interacting with other transcription factors to increase transcription (e.g., HIF1α, STAT3, and CBP/P300)^87^. Similarly, the NTHL1^88^, PCNA^89^, KRas^90^, and HRas^91^ promoters harbor PQS, of which the 8-oxo-dG modification potentially increases transcription. In contrast, 8-oxo-dG formation in G-quadruplex represses transcription, possibly through topological changes of the G-quadruplex, as shown in reporter genes with the RAD17^92^ and NEIL3^93^ promoters. Moreover, synthetic 8-oxo-dG, which was introduced into the template strand of PQS, produces transcriptional repression, which is shown on the VEGF gene promoter^94^.

In addition to the G-quadruplex, the regulatory binding site of the NF-kB transcription factor interacts with 8-oxo-dG together with OGG1 to contribute to transcriptional activation (Fig. 2c). This was observed in ROS production and subsequent 8-oxo-dG modification during ligand-induced gene activation, particularly in TNFα-responsive NF-kB target genes^84^. During the exposure of cells to TNFα, 8-oxo-dG recruits OGG1 upstream of NF-kB binding sites located in promoter regions of proinflammatory genes (e.g., TNFα, CCL20, CXCL1, B2M, IL1B, and CXCL2)^84,95,96^. Regardless of the enzymatic activity, promoter-associated OGG1 increases the occupancy of NF-kB, facilitates the assembly of the transcriptional machinery, and finally activates the transcription of target genes^84,95,96^. As OGG1 searches for 8-oxo-dG through rotational diffusion and introduces a bend in the DNA duplex^97,98^, the OGG1-increased recruitment can be mediated by inducing allosteric changes in the chromatin that create an interface for transcription factor binding (e.g., NF-kB and estrogen receptor). Moreover, hypoxia-induced genes, including VEGF, have also been found to recruit OGG1 and APE1 to their oxidized promoter regions, thereby facilitating the binding of hypoxia-inducible factor-1α (HIF1α) to the responsive element to increase their transcription^83^. Intriguingly, in response to oxidative stress, multiprotein complexes, including OGG1, APE1, Ku70, and RNA pol II, are recruited to negative calcium responsive elements (nCaRE) and activate the transcription of the sirtuin-1 (SIRT1) deacetylase, indicating their involvement with other epigenetic regulations^86^.

### Crosstalk with histone modifications

8-Oxo-dG is associated with histone demethylation, wherein DNA oxidation is induced by local ROS generated during the demethylation reaction and subsequently bound by OGG1, which mediates transcriptional regulation (Fig. 2d). This regulation was initially observed in the activation of the estrogen receptor, which led to the transcriptional activation of its target genes (e.g., BCL2 and TTF1) in breast cancer cells^85^. Upon estrogen treatment, the estrogen receptor binds to the promoter of the target gene and activates the resident histone lysine-specific demethylase (LSD1, also known as KDM1A) to remove H3K9me2, which is associated with transcriptional repression^85^. During this enzymatic reaction, H_2_O_2_ is produced as a byproduct in the nucleus and oxidizes local DNA to produce 8-oxo-dG, which interacts with repair proteins. OGG1 recruits transcription factors, and APE1 induces nicks in the DNA to be assembled by topoisomerase IIβ (TOPIIβ), eventually triggering chromatin conformation changes and transcriptional activation.

A similar mechanism was observed for the demethylation of another histone, H3K4me2, upon activation of the Myc transcription factor^99^. During tumor transformation, hyperactivated Myc occupies its responsive element E-box on the promoter of critical target genes (e.g., nucleolin and carbamoyltransferase-dihydroorotase) and activates LSD1 to demethylate H3K4me2 with the production of H_2_O_2_^99^. Then, local oxidation of DNA is triggered and bound by OGG1 and APE1, thus facilitating the activation of transcription. Likewise, in prostate cancer cells, activation of androgen receptors mediates the increased transcription of the target gene by following the serial activation of the androgen receptor, monoamine oxidase LSD1, H3K4me2 demethylation, H_2_O_2_ production, local 8-oxo-dG oxidation, OGG1-APE1 recruitment, and transcription of androgen-induced target genes, including miRNAs (e.g., KLK3, TMPRSS2, miR-125b2, and miR-133b)^100^. In addition, during TGFβ-induced target gene activation, 8-oxo-dG oxidation, generated by ROS production during histone demethylation and ligand activation, is required to direct target gene transcription for epithelial-to-mesenchymal transition (EMT)^101^. In this regulation, upon initial activation of phosphorylated SMAD2/3, the regulation axis of LSD1-H_2_O_2_-DNA oxidation-OGG1-APE1 is activated for transcription of EMT genes (e.g., SNAI1 and WIF1)^101^. The second oxidative wave from TGFβ stimulation further accumulates 8-oxo-dG and the OGG1 complex, thereby guiding the formation of repressive complexes (LSD1, HDAC3, NcoR1, and newly synthesized SNAI1, which silence target genes)^101^.

### Crosstalk with DNA methylation

For DNA cytosine methylation (5-methylcytosine, 5mC), 8-oxo-dG reduces the binding affinity to DNA methyltransferases (DNMTs), thereby inhibiting the methylation of CpG islands as part of a passive mechanism^102–104^ (Fig. 2e). Furthermore, 8-oxo-dG occurs in methylated CpG islands and interferes with the binding of methyl-CpG binding proteins (MBPs), including MeCP2, thereby inhibiting their transcriptional suppression activity^105^. Moreover, even in cases where 8-oxo-dG is not directly formed in the DNA methylation sites, demethylation of adjacent 5mC is stimulated, as shown in some cancer cells (e.g., prostate cancer)^106^. In this oxidative stress-induced DNA demethylation, OGG1 has essential roles in recognizing 8-oxo-dG lesions and recruiting TET1, which can oxidize adjacent 5mC to 5-hydroxymethylcytosine (5hmC) for DNA demethylation^107^ (Fig. 2e). Similar to histone demethylation, CpG island demethylation by TET1 produces nuclear ROS. Therefore, resident DNA can be oxidized to 8-oxo-dG and bound by OGG1, thus directing target gene transcription via the oxidative DNA damage response, as shown in TNFα ligand-induced gene activation^108^ (Fig. 2e). In contrast, 8-oxo-dG DNA oxidation has been reported to contribute to the DNA damage response mechanism, which suppresses the expression of damaged regions by inducing CpG island methylation and chromatin silencing^109^. 8-Oxo-dG-bound OGG1 interacts with chromodomain helicase DNA-binding protein 4 (CHD4) to recruit repressive chromatin proteins (EZH2 and G9a) and DNA methyltransferases (DNMT1, DNMT3A, and DNMT3B)^110^. Indeed, ROS induce hypermethylation of the E-cadherin promoter by increasing Snail expression via recruitment of HDAC1 and DNMT1 in hepatocellular carcinoma^111^. Although the effect of 8-oxo-dG on DNA methylation may depend on the different contexts of promoter sequences and cellular status, further study is required to clarify the general effect of 8-oxo-dG on DNA methylation.

## Sequencing of 8-oxoguanine in the genome

Although various epigenetic functions of 8-oxo-dG have been observed in biochemical studies, the genome-wide distribution of 8-oxo-dG should be determined to conclusively understand the interplay between 8-oxo-dG and other epigenetic modifications^112^. Initially, using an 8-oxo-dG antibody, fluorescence in situ detection of 8-oxo-dG (~1000 kb resolution) in metaphase chromosomes (human peripheral lymphocytes) revealed that 8-oxo-dG is unevenly located within the preferred regions of recombination and single-nucleotide polymorphism^113^. Subsequently, 8-oxo-dG-containing DNA fragments in the rat kidney genome were identified by immunoprecipitation followed by microarray analysis (approximately 10 kb resolution)^114^. This study showed that 8-oxo-dG predominantly occurs within gene deserts in correlation with lamina-associated domains (LADs), suggesting that the genome in the nuclear periphery might be spatially prone to oxidative damage.

Recently, several high-throughput sequencing methods have been developed to map 8-oxo-dG in the genome (Table 2) and have revealed that the distribution of 8-oxo-dG is heterogeneous and not uniformly detected throughout the genome^112^. Using selective biotin conjugation of 8-oxo-dG under mild oxidation, genomic fragments with biotin-labeled 8-oxo-dG were isolated and sequenced (OG-Seq) in mouse embryonic fibroblasts (MEFs in the OGG1 null background) and found to be enriched in the promoter, 5′UTR, and 3′UTR regions relative to the expected frequency^115^. To investigate 8-oxo-dG at single-nucleotide resolution, Click-code-seq was developed, which analyzed the locations of barcode sequences after ligation to Click-dGTPs that were incorporated into 8-oxo-dG excision sites prepared by in vitro treatment of repair enzymes (Fpg and APE1)^116^. In the yeast genome, Click-code-seq revealed that 8-oxo-dG accumulates at sites of high nucleosome occupancy compared to nucleosome-free linker regions. Based on OG-Seq, CLAPS-seq (chemical labeling and polymerase stalling sequencing) was developed for single-nucleotide resolution, which relies on the feature that DNA polymerase stalls before biotin-labeled 8-oxo-dG^117^. In its application to a human HeLa cell genome, CLAPS-seq showed that 8-oxo-dG is underrepresented in the G-quadruplex and promoter sequences with high GC content^117^.

**Table 2:** Genome-wide 8-oxo-dG-sequencing methods.

	OG-Seq^115^	Click-code-seq^116^	CLAPS-seq^117^	AP-Seq^118^	OxiDIP-Seq^119,120^	enTRAP-Seq^121^	ChIP-Seq^122^
Prerequisite	- 8-oxo-dG-specific in vitro oxidation- biotin conjugation	- In vitro digestion with Fpg and APE1 - Incorporation of Click-dGTP - Biotin conjugation with code sequences	- 8-oxo-dG-specific in vitro oxidation - Biotin conjugation	- In vitro digestion with OGG1 - ARP conjugation	- None	- None	- Formaldehyde treatment for crosslinking
8-Oxo-dG isolation	Streptavidin-biotin interaction	Streptavidin-biotin interaction	Streptavidin-biotin interaction	Streptavidin-biotin interaction	Immunoprecipitation with 8-oxo-dG antibody	Affinity purification with K249Q hOGG1 protein	Immunoprecipitation with OGG1 and APE1 antibodies (acetylated active forms)
Analysis of 8-oxo-dG distribution	Peak analysis of compiled reads	Nucleotide after code sequence	Peak analysis of compiled reads and polymerase stalling site	Peak analysis of compiled reads	Peak analysis of compiled reads	Peak analysis of compiled reads	Peak analysis of compiled reads
Estimated resolution	~150 bp	Single-nucleotide	Single-nucleotide	~250 bp	~200–800 bp	~100–1000 bp	Size of DNA fragmentation
Applied genome	Mouse embryonic fibroblast (MEF)	*S. Cerevisiae* (Yeast)	HeLa	X-ray irradiated HepG2	MEF^119^ and MCF10A^119,120^	MEF	A549 and HCT116
Results from 8-oxo-dG sequencing	- Enriched in regulatory elements (promoter, 5′UTR and 3′UTR)	- Enriched in regions with high nucleosome occupancy	- Underrepresented in G-quadruplex and promoter	- Enriched in retrotransposons and microsatellites, open chromatin regions and G-quadruplex; Underrepresented in closed chromatin regions	- Enriched in DNA damaged regions (double strand breaks)^119^, promoter regions (enriched in repair proteins, G-quadruplex, CG skew, and bidirectional transcription)^120^	- Enriched in open chromatin regions and regulatory elements (promoters, CpG islands, and 5′UTR)	- Located in putative quadruplex sequences (PQS)

As 8-oxo-dG can be converted to AP sites by OGG1 treatment, a method called AP-Seq was developed by conjugating AP sites using a biotin-labeled aldehyde reactive probe (ARP) for affinity purification and sequencing in X-ray-irradiated HepG2 cells^118^. The AP sites containing 8-oxo-dG are largely accumulated in retrotransposons (long interspersed elements (LINEs) and short interspersed elements (SINEs)) and microsatellites and are generally abundant in open chromatin features (e.g., H3K4me3 and H3K9ac), correlated with GC content, but deficient in closed chromatin (e.g., H3K9me3)^118^. In particular, 8-oxo-dG-derived AP sites are relatively more abundant in G-quadruplex sequences than in total AP sites^118^.

High-throughput sequencing of DNA fragments isolated by immunoprecipitation with an 8-oxoguanine antibody was developed (OxiDIP-Seq) and applied to human and mouse genomes (MCF10A and MEFs)^119^. Approximately 42% of the 8-oxo-dG peaks identified were localized at gene loci and correlated with the activation of the DNA damage response (DDR) with double strand breaks (e.g., H2AX ChIP-Seq)^119^. Within human gene loci, 8-oxo-dG peaks accumulated in promoter regions with repair proteins (OGG1 and PARP ChIP-Seq) and prevailed in G-quadruplex, CG skew, and bidirectional transcription. As 8-oxo-dG peaks are reduced in the genome of quiescent (G_0_) cells^120^, 8-oxo-dG accumulation seemed to depend on DNA replication and/or transcription. Similarly, using enTRAP-Seq, which employs a catalytically inactive OGG1 mutant (K249Q) to isolate 8-oxo-dG lesions, OGG1-bound 8-oxo-dG is enriched in open chromatin regions and regulatory elements (e.g., promoters, CpG islands, and 5′UTR)^121^.

In addition, 8-oxo-dG sequencing based on the binding sites of hyperactivated OGG1 (acetylated OGG1 ChIP-Seq) was recently attempted together with sequencing AP sites (AP-Seq), activated APE1 binding sites (APE1 and acetylated APE1 ChIP-Seq; repair-seq), and G-quadruplex sequences (G4 ChIP-Seq using G-quadruplex-specific antibody, BG4) in cancer cell lines (A549 and HCT116)^122^. This comparative analysis revealed that 8-oxo-dG modification and the subsequent AP1 site with APE1 binding were required to form G-quadruplex structures in the genome, which coincided with the results of previous biochemical studies^112,122^.

As described above, the genome-wide distribution of 8-oxo-dG was examined to yield insights into the global nature of 8-oxo-dG within the genome. Indeed, tracking the distribution of 8-oxo-dG is essential for understanding the general mechanisms that regulate gene expression and redox-dependent pathogenesis. The development of various sequencing methods for 8-oxo-dG has set up stages to further inspect the epigenetic roles of 8-oxo-dG at the genome-wide level in conjunction with other interacting repair proteins, functional elements, and epigenetic modifications. However, diverging conclusions have been drawn from distinct methodologies and perspectives. Therefore, the distribution of 8-oxo-dG in the genome still needs to be clarified with further development of precise sequencing methods and analyses.

### 8-OXOGUANINE IN RNA

#### o^8^G

Compared with the studies on 8-oxo-dG, there are only a few studies on o^8^G, and its repair mechanisms and regulatory functions are largely unknown. Although both DNA and RNA can react with ROS, the unique characteristics of RNA make it vulnerable to oxidation^16^. This is probably because RNA is more reactive (2’-hydroxyl group), exposed (single-strand, absence of protein protection, such as histones, and cellular location in the vicinity of ROS production), and unsecured (lack of redundant repair systems) than DNA^11^. Numerous forms of oxidized RNA are generated, analogous to oxidized forms in DNA (e.g., o^8^G, 8-oxoadenine, 5-hydroxyuridine, and 5-hydroxycytidine)^12,123^. Among them, o^8^G is the most abundant product and is susceptible to further oxidation, strand breakage, and base removal. However, o^8^G has drawn less attention because of the rapid turnover of the RNA molecule^124^. Nevertheless, not every RNA is unstable: a considerable number of RNAs have long half-lives, as documented for rRNA and tRNA, which even last for several days^125^. RNA plays a wide range of biological roles from imparting genetic information to regulating gene expression; thus, RNA oxidation can critically lead to miscellaneous dysfunctions and regulation of both coding and noncoding RNAs, which are related to pathophysiological consequences under oxidative stress^11,126^. Therefore, o^8^G is not only involved in oxidative damage but also serves as an epitranscriptional modification, as we have discussed for 8-oxo-dG.

#### o^8^G in diseases

Initially, o^8^G was detected using HPLC separation coupled with electrochemical measurement, in which the amount was higher than that of 8-oxo-dG in the study of hepatocarcinogens (e.g., 2-nitropropane) in rats^127^. Under normal conditions, 8-oxoguanine and its derivatives, including o^8^G, were selectively detected in human blood using an 8-oxoguanine-specific antibody^128^. These early reports suggested the occurrence of o^8^G, which could be relevant to biological phenotypes, as observed in 8-oxo-dG. Later, o^8^G was confirmed to occur in cytoplasmic RNAs by observing retained 8-oxoguanine immunostaining in DNase I-treated brains of patients with Alzheimer’s disease (AD)^129^, which is associated with increasing ROS. o^8^G was also identified in the brains of patients with Parkinson’s disease^130^ and aged mouse brains^131^, wherein the quantity of o^8^G correlated with memory loss and mitochondrial decay, which could be partially reversed by antioxidant treatment^132^. Based on these observations, the oxidative modification of cytoplasmic RNAs, which may include mRNA, rRNA, tRNA, and miRNA, has been proposed to function in redox-related disease phenotypes, especially in the case of neurodegenerative disorders. In addition to the focus on neuronal diseases (e.g., AD, Parkinson’s disease, amyotrophic lateral sclerosis (ALS), spinal cord injury, epilepsy, dementia of Lewy bodies, prion disease, and subacute sclerosing panencephalitis)^11^, o^8^G-RNA has been investigated in many other diseases, such as atherosclerosis, Down syndrome, hepatocarcinogenesis, xeroderma pigmentosum, hereditary hemochromatosis, disuse atrophy, rimmed vacuole myopathy, emphysematous lungs, chronic obstructive pulmonary disease, and aging^126^; however, little is known about the direct causality and underlying mechanisms despite its potential dysfunction.

## Epitranscriptional roles of 8-oxoguanine

### o^8^G-mRNA-mediated defective protein synthesis

**Fig. 3:**
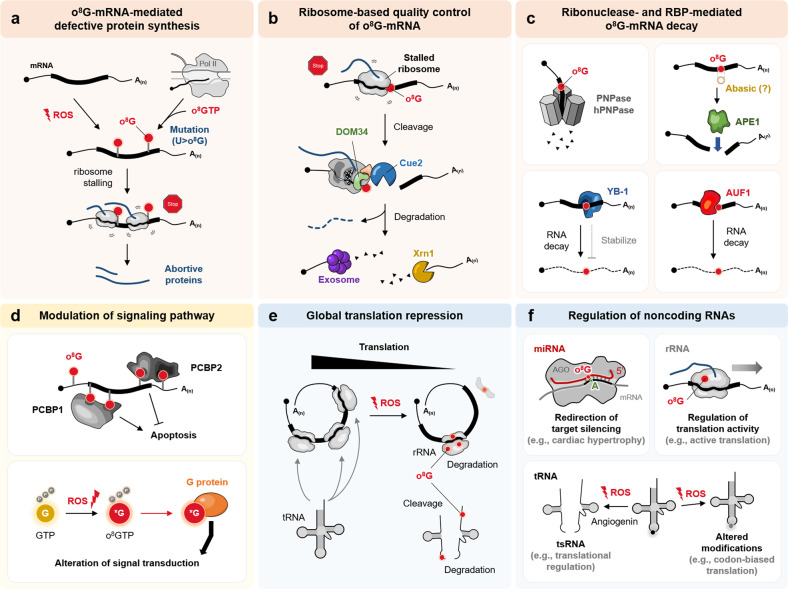
Epitranscriptional roles of o^8^G. **a** Defective protein synthesis induced by o^8^G. ROS-induced o^8^G in mRNA lowers its coding capacity and causes ribosome stalling, thus advertently producing abortive proteins. ROS can oxidize the free ribonucleotide o^8^GTP, which can be incorporated during RNA transcription and cause U > o^8^G mutation in mRNA. **b** Ribosome-based quality control of o^8^G-mRNAs. o^8^G-induced ribosome stalling in mRNA triggers NGD, of which the complex consists of DOM34 and Cue2, cleaves o^8^G-mRNAs, and induces degradation using decay machinery, comprising exosomes and Xrn1. **c** o^8^G-mRNA degradation mediated by ribonucleases and RBPs. PNPase binds o^8^G and degrades o^8^G-containing RNAs. APE1 binds o^8^G or o^8^G-derived abasic sites to recognize oxidized RNAs and triggers cleavage for degradation. YB-1 interacts with o^8^G and induces RNA decay or stabilization. **d** o^8^G modulates signaling pathways by interacting with RBPs and G proteins. PCBP1 and PCBP2 recognize heavily oxidized RNAs by binding to two o^8^Gs sites, leading to the opposite effects; PCBP1 activates but PCBP2 inhibits apoptotic signaling (upper panel). ROS induce oxidation of free ribonucleotide (o^8^GTP), which binds to G protein and modulates the function in signal transduction (lower panel). **e** o^8^G-induced global repression of translation. Under ROS production, o^8^G modification, which predominantly occurs in rRNA and tRNA, induces decay and cleavage of oxidized rRNA and tRNA, resulting in the global suppression of translation. **f** o^8^G regulates the functions of noncoding RNAs. Position-specific o^8^G in the seed region of miRNAs redirects its target recognition via o^8^G•A base pairing, resulting in the induction of pathophysiological changes (e.g., cardiac hypertrophy induced by o^8^G-miR-1; upper left panel). Position-specific o^8^G in rRNA may regulate translational activity (upper right panel). Oxidative stress-induced cleavage of tRNA may be regulated by o^8^G, generating tsRNAs as regulators of the stress response. o^8^G modification in tRNA may result in changes in other modifications, which lead to alterations in tRNA function (lower panel).

In the analysis of RNA oxidation, cytoplasmic RNAs undergo pathological oxidation (as in neurodegenerative disorders), and o^8^G modification occurs in mRNA derived from the brains of patients with AD^133^ and ALS^134,135^. Substantial amounts of poly(A)-tailed mRNAs were found to contain o^8^G with variable susceptibility depending on mRNA species relative to low oxidation in normal brains^136^. Some mRNAs were more sensitive to o^8^G oxidation than other transcripts, regardless of their abundance. mRNA oxidation appeared to be an early event that immediately occurs after ROS generation, preceding cell death in primary cortical neurons^135^ and neuronal degeneration in a familial ALS mouse model (SOD1^G93A^)^134^. This evidence suggests that the selective oxidation of some mRNAs can mediate the biological consequences of oxidative stress. Oxidized mRNAs cause a reduction in protein synthesis and defective translation with errors^137^; oxidized reporter mRNAs, generated in vitro, are not well expressed and are often translated into defective proteins with aggregation^133,135^. Indeed, o^8^G in mRNA can deteriorate codon-anticodon interactions and suppress translation^12^. Furthermore, o^8^G in mRNA lowers the quality of genetic information, which leads to ribosome stalling and the synthesis of impaired proteins via altered base pairing (o^8^G•A)^123^. The oxidized reporter mRNA produces abortive short peptides, which are speculated to be caused by premature termination of the translational process and/or rapid proteolytic degradation of error-containing proteins^137^. However, o^8^G in codons has recently been reported to cause little to no o^8^G•A miscoding but to stall ribosomes by reducing the rate of peptide-bond formation during translation^135^. Therefore, abortive proteins from o^8^G-mRNA seem to be mainly generated by premature termination of translation caused by ribosome stalling (Fig. 3a).

The o^8^G in mRNA seems to be generated by a direct oxidative reaction on guanine, but it can be alternatively synthesized during mRNA transcription by incorporating oxidized o^8^GTP into RNA polymerase (Fig. 3a), as observed by the adduct of 8-oxo-dGTP during DNA replication^7^. Although the rate of o^8^GTP incorporation is low in humans (~2%)^138^, the pool size of ribonucleotides is much larger than that of 2’-deoxyribonucleotides, and as a consequence, o^8^G modification is more likely to be incorporated into mRNA^123^. In support of this hypothesis, the o^8^G adduct was detected at much higher concentrations in RNA relative to the 8-oxo-dG adduct in DNA, as measured by treating the human lung epithelial cell line with isotope-labeled H_2_O_2_^139^. To prevent the o^8^G adduct, MTH1 and NUDT5 proteins in humans degrade o^8^GTP and o^8^GDP to o^8^GMP, which is unusable for RNA synthesis^138,140^. MTH1 and NUDT5 also hydrolyze 8-oxo-dGTP and 8-oxo-dGDP, thus preventing the misincorporation of oxidized nucleotides into DNA and mRNA. In line with this, o^8^GTP treatment in MTH1-knockdown cells drastically increased the o^8^G mRNA content^73^. It should be noted that the incorporation of o^8^G into the nucleotide position, where it should be U for base pairing with A during mRNA transcription, can direct transcriptional mutation, resulting in the suppression of a nonsense mutation in the luciferase reporter gene by triggering U > o^8^G changes in its mRNA sequences (Fig. 3a). While o^8^G in this reporter system represents a possible o^8^G-directed editing of mRNA transcripts that leads to changes in protein sequence, o^8^G in mRNA also shows advertent translation to pathogenic proteins, while excessive o^8^G in mRNA induces the accumulation of aggregable amyloid β peptides in cells expressing amyloid precursor proteins^73^. Although it still remains elusive, the o^8^G adduct in mRNA is speculated to specifically induce mutations during transcription and ribosome stalling, which can be used as a regulatory mechanism for selectively producing defective proteins in response to the cellular redox status.

### Ribosome-based quality control of o^8^G-mRNA

o^8^G in mRNA is highly deleterious to its coding ability, as it causes ribosome stalling and subsequently generates abortive proteins, which increases cytotoxicity and deteriorates ribosome homeostasis^12,123^. In the cell, potentially deleterious RNAs containing chemical damage or premature termination codons are subjected to RNA surveillance pathways, which monitor the quality of RNAs and limit the use of aberrant ones by inducing RNA decay, chemical modification, localization, and sequestration^141^. Although RNA surveillance of oxidized mRNA has not been extensively investigated, recent studies have shown that o^8^G-mRNAs activate no-go decay (NGD) by stalling ribosomes (Fig. 3b). In a defined in vitro system, a single modified o^8^G in the codon was shown to cause ribosome stalling by disrupting tRNA selection regardless of the position^142^, wherein its frequent *syn* conformation on ribosomes and potential pairing with adenosine cannot sufficiently promote the required conformational changes to proceed with peptide-bond formation^143^. Then, the activated NGD degrades target RNA using decay machinery (5′-3′ Xrn1-dependent and 3′-5′ exosome-dependent degradations) after inducing endonucleolytic cleavage (e.g., Cue2^144^). o^8^G-mRNA accumulates in the absence of NGD factors in yeasts (Dom34p and Xrn1p)^142^. Notably, recent in vitro assays showed that Xrn1 stalls at the o^8^G sites, suggesting the presence of other factors that contribute to the decay of oxidized RNA^145^. Concomitantly, the associated ribosome quality control is activated, depending on LTN1 and Hel2 expression with oxidation and alkylation damage agents in yeasts^146^. Therefore, incomplete nascent peptides and stalled ribosomes can be removed and dissociated, which is necessary for avoiding the toxicity of aberrant proteins and rescuing diminished translation capacity. Although ribosome-based quality control exists to secure the proper coding capacity of mRNA and in part functions for o^8^G-mRNAs, rapid degradation of o^8^G-mRNAs may be used to selectively repress their expression depending on the redox state (Fig. 3b). The possibility of redox-mediated regulation remains to be elucidated in mammalian cells, but to some degree, it has been shown with in vitro-generated mRNAs that one o^8^G adduct in the coding sequence can destabilize mRNA in human cells^146^.

### Ribonuclease- and RBP-mediated o^8^G-mRNA degradation

In an attempt to identify regulatory proteins for o^8^G, polynucleotide phosphorylase (PNPase), an exoribonuclease in the RNA degradosome complex^147^, was isolated as an interacting partner of o^8^G-RNA; its overexpression protected *E. coli* from oxidative stress^148^. Human PNPase (PNPT1) binds o^8^G^149^, functions in the mitochondria for its homeostasis^150^, reduces RNA damage, and induces tolerance to oxidative stress^151^ (Fig. 3c). Increased PNPase binding to o^8^G, designed by computational evolution, renders cells resistant to H_2_O_2_ treatment^152^. This evidence suggests that PNPase can directly recognize o^8^G-RNA and trigger its degradation to tolerate oxidative stress. In addition, a DNA repair enzyme for BER, APE1, has been proposed to function in o^8^G degradation^153^. APE1 can cleave abasic single-stranded RNA^154^ and regulate c-Myc mRNA levels in tumor cells^155^, implying its putative nonrepair role in regulating posttranscriptional gene expression. Supporting APE-mediated o^8^G degradation, APE1 knockdown increases o^8^G levels of total RNA and rRNA in H_2_O_2_-treated HeLa cells^156^. In addition, APE1 functions in ribosome biogenesis and RNA processing by interacting with several protein partners, such as nucleophosmin and nucleolin^156^. However, it is still questionable whether APE1 directly reacts with the o^8^G site because APE displays marginal activity on the o^8^G substrate relative to abasic DNA, even though the substrate is artificially generated by embedding o^8^G into DNA^157^. Therefore, APE1 may act on o^8^G-derived abasic sites, which are processed either by RNA glycosylases such as MPG^158^ or by subsequent oxidative reactions that lead to hydrolysis (e.g., oxidative depurination of o^8^G, facilitated by cytochrome c)^159^ (Fig. 3c). It was recently reported that APE1 destabilizes abasic mRNAs derived from ROS-generating mitochondria, which function in oxidative phosphorylation^160^.

Several RNA-binding proteins (RBPs) have been identified with o^8^G. Y-box binding protein 1 (YB-1, also called YBX) preferentially binds o^8^G-containing RNAs through its cold shock domain (Fig. 3c), and its overexpression in *E. coli* confers tolerance against oxidative stress^161^. However, the detailed mechanism and consequences of this interaction remain unknown. It has been proposed that YB-1 likely triggers RNA decay by recruiting other ribonucleases as components of processing bodies. Otherwise, YB-1 can stabilize o^8^G-containing mRNAs by conferring RNA-chaperone function^162^, preventing the decapping process^163^, or sequestering the mRNAs into stress granules for protection under oxidative stress^164^. Through mass spectrometry of o^8^G-interacting proteins, AU-rich element RNA-binding protein 1 (AUF1, also called HNRNPD) and HNRNPC were identified in HeLa cells, wherein knockdown of these genes increases sensitivity to oxidative conditions^165^. Among them, AUF1 can destabilize o^8^G-containing mRNA^166^, consistent with its known function to promote mRNA decay by binding to AU-rich elements^167^ (Fig. 3c). Oxidized mRNA increases in AUF1-deficient human cells^166^. Overall, o^8^G is recognized directly by ribonucleolytic enzymes (PNPase and APE1) and RBPs (YB-1 and AUF1) as part of ribosome-independent mRNA quality control, but o^8^G can be used as an epitranscriptional modification that marks selective mRNA degradation in terms of posttranscriptional gene repression (Fig. 3c).

### Modulation of the signaling pathway

Another RBP, poly(C)-binding protein 1 (PCBP1, also called HNRNPE1), was identified using oligoribonucleotides containing two o^8^Gs and mass spectrometry^168^. Intriguingly, PCBP1 only binds heavily oxidized RNAs through its two RNA-binding KH domains and does not destabilize target mRNAs but instead activates signaling pathways that lead to apoptotic cell death^168^ (Fig. 3d). Decreased caspase-3 activation and PARP cleavage are observed in the absence of PCBP1. Based on this, PCBP1 binding to excessive o^8^Gs was suggested to initiate a damage-signaling pathway that leads to apoptosis under oxidative stress. In contrast, even though PCBP2 binds heavily oxidized o^8^G-RNA, PCBP2 suppressed ROS-mediated cell death^169^ (Fig. 3d). This opposite effect is hypothesized to be caused by counteracting PCBP1 but remains elusive. In addition, cytochrome c interacts with o^8^G and has been proposed to induce apoptosis by catalyzing o^8^G oxidation to depurination and crosslinking, which may facilitate cytochrome c release from mitochondria^159^. Furthermore, increased concentrations of free o^8^GTP during oxidative stress have been reported to modulate the activity of small G proteins (Fig. 3d). o^8^GTPγS, an oxidized unhydrolyzable analog of GTP, activated Ras-ERK pathways in vitro more than its unoxidized form^170^ but inactivated Rac1 and NADPH oxidase (NOX)^171^. Consistently, o^8^GTPγS inhibits Rac1 activation and NOX-derived ROS production, resulting in the downregulation of inflammatory neutrophil activation^171^. Similarly, exogenous 8-oxo-dGTP was shown to inhibit Rac1 and NOX^172^, ameliorating various inflammation-related diseases^173^. Under certain conditions, o^8^G modulates several signaling pathways through o^8^G-RNA or free o^8^GTP, which induces apoptosis and suppresses inflammatory responses^16^. Further studies are required to elucidate the detailed roles and mechanisms of o^8^Gs in modulating signaling pathways.

### Global translation repression

To properly respond and adapt to redox changes, the suppression of global translation should be precisely and timely controlled to initiate the synthesis of new stress-defensive proteins. o^8^G modification appears to have a negative effect on gene expression, decreasing the efficiency and fidelity of translation^174,175^. o^8^G in rRNA was dramatically increased in H_2_O_2_-treated *E. coli*, and the folding structure of rRNA and tRNA did not protect their oxidation in vitro^176^. In the brains of patients with AD, ribosome dysfunction is associated with increased RNA oxidation as an early event, resulting in a decreased rate and capacity for protein synthesis^177^. This implies that the overall translation can be immediately reduced by producing an o^8^G modification in the translational machinery, which may be actively used to hold translation with regard to oxidative stress and lead to pathophysiological changes (Fig. 3e).

rRNA has been proposed to be sensitive to ROS levels by interacting with iron. In neurons of patients with AD, rRNA is bound by redox-active iron (Fe^2+^, iron(II))^178^, which is competent to produce reactive hydroxyl radicals (Fenton reaction with H_2_O_2_) and hence readily oxidized to o^8^G. Oxidized ribosomes show a significant reduction in translation^179^. Ribosome activities in translation largely depend on precisely tuned conformational transitions within the rRNA folding framework. Therefore, o^8^G modification potently perturbs the critical rRNA structure by disrupting existing interactions and/or rearranging new structures via altered base pairing (o^8^G•A)^13^. In line with this, mitochondrial rRNA sequences are evolutionally shifted to minimize guanines located in exposed surfaces^180^ as well as the overall RNA content in the ribosome^181^, converging into a more protein-based architecture. This is presumably driven by cellular fitness to eliminate potent o^8^G sites in the ribosome because they are vulnerable to ROS generation during mitochondrial energy production.

The impairment in ribosome function correlates with reduced rRNA and tRNA levels, as observed in the brains of patients with AD and many other cases^177^. This is likely caused by RNA quality control, which detects and degrades defective rRNAs and modification-deficient tRNAs^182^ through nonfunctional rRNA decay (NRD)^183,184^ and rapid tRNA decay (RTD)^185^ pathways. Intriguingly, the recognition of RTD for tRNA modification depends on the overall stability of the tertiary structure^186^, implying that o^8^G may be involved in this process by inducing unconventional base pairing (o^8^G•A). Moreover, tRNA undergoes specific cleavage in response to oxidative stress^187–189^, resulting in the downregulation of functional tRNA pools that can limit the translational elongation process^187^. Considering that the majority of RNA in cells consists of rRNA and tRNA, the overall direction of o^8^G modification would be the suppression of global translation to the extent that it relies on the cellular redox state (Fig. 3e).

### Regulation of noncoding RNAs

Beyond damage signals from extensive o^8^G oxidation, position-specific o^8^G can serve as an epitranscriptional modification that alters regulatory RNA–RNA interactions via o^8^G•A base pairing^13^, which is particularly important for noncoding RNAs in response to redox changes. Recently, o^8^G oxidation has been observed in miRNAs, which are regulatory noncoding RNAs that recognize hundreds of target mRNAs through base pairing to their seed regions (positions 2–8) and suppress their expression by reducing mRNA stability and/or translation^190^. Depending on the functions of their targets, miRNAs have diverse pathophysiological roles. For this reason, any change in the seed sequence can alter different sets of target transcripts^191^, resulting in the redirection of miRNA-mediated functions (Fig. 3f). Indeed, extensive oxidation of miR-184 has been reported to target Bcl-xL and Bcl-w, thereby increasing cardiomyocyte cell death and ischemia–reperfusion (IR) injury^192^, implicating that oxidized damage in miRNA can alter its biological function.

Most certainly, o^8^G modification in cardiac miRNAs has been precisely identified by developing the o^8^G sequencing method (o^8^G-miSeq), which isolates o^8^G-miRNA by immunoprecipitation with an 8-oxoguanine-specific antibody and determines o^8^G positions at single-nucleotide resolution by analyzing the o^8^G > T mutation in cDNA^193,194^. Under oxidative hypertrophic conditions, o^8^G is generated predominantly at position 7 of miR-1 (7o^8^G-miR-1), which results in the redirected recognition and silencing of target genes. This effect is dependent on o^8^G•A base pairing because substitution of o^8^G with U at position 7 of miR-1 (7U-miR-1) causes the cardiac phenotype in transgenic mice. Furthermore, antagonizing 7o^8^G-miR-1 using its sponge inhibitor (competitive target sites of o^8^G•A base pairing) prevented cardiac hypertrophy in mice, demonstrating that 7o^8^G-miR-1 serves as an endogenous driver of related pathogenesis. As cardiac hypertrophy is not the only disease involving ROS, other redox-associated conditions, such as tumors, can be regulated by the o^8^G modification of miRNAs. Additionally, o^8^G and its associated proteins can function in miRNA processing. APE1 recognizes o^8^G-derived abasic RNAs, mediating the processing of miR-221/222 through its endonuclease activity and interaction with a component of the microprocessor Drosha, which are enhanced by oxidative stress^195^. Therefore, APE1 consequently induces repression of miR-221/222 target genes, including the tumor suppressor PTEN, in cancer cells. An o^8^G binding protein, PCBP1, modulates miRNA processing as a component of the miRNA-processing pathway that regulates miRNA biogenesis in myoblasts, resulting in the control of skeletal muscle differentiation^196^.

Although excessive oxidation of rRNA and tRNA tends to cause global repression of translation, specific o^8^G oxidation can exert regulatory roles in functional transition. In H_2_O_2_-treated *E. coli*, o^8^G was predominantly identified in the large subunit of the ribosome by o^8^G immunoprecipitation and sequencing^197^. Generally, oxidation in ribosomes inhibits their activity, but the oxidation of a specific position in the active site surprisingly facilitates translation (Fig. 3f). While this experiment used oxidized nucleotides instead of o^8^G, this study suggests the importance of positional oxidation in terms of functional transition in rRNA. Regarding tRNA oxidation, since tRNA already contains various base modifications, oxidation in tRNA^189^ appears to alter other modifications^198^, including redox-sensitive sulfur-containing nucleotides, such as 2-thiouridine (S2U), rather than generating o^8^G. Reprogramming of tRNA modification mediates codon-biased translation (TTG codon, recognized by increased cognate tRNA), which occurs in yeast under oxidative stress^199^ (Fig. 3f). In addition, oxidative stress has been shown to induce tRNA cleavage^187^ through specific enzymes (e.g., angiogenin in humans^200^, Rny1 in yeasts^188^) to confer specific regulation and function, not just generated as byproducts of oxidative damage (Fig. 3f). tRNA fragments (tRNA-derived small RNAs; tsRNAs) promote cell death^188^ and stress granule assembly, suppress translational initiation^201^, and induce RNA-mediated silencing, similar to miRNAs. Furthermore, CCA deactivation, a cleavage of the conserved 3-CCA termini of tRNAs by angiogenin, was observed to be rapidly induced by oxidative stress but quickly restored by the CCA-adding enzyme to reactivate translation in the absence of ROS^202^. Under mild oxidative stress, rRNA undergoes site-specific cleavage, which may regulate a specific function^203^. However, there is still a lack of direct evidence that o^8^G is involved in this regulation. Therefore, further studies are needed to determine the relationship between noncoding RNAs and o^8^G modification.

## Concluding remarks

To date, 8-oxoguanine has been mainly described as a product of oxidative damage, but growing evidence has highlighted that 8-oxoguanine can function as an epigenetic (Fig. 2) and epitranscriptional modification (Fig. 3). Such features seem to be intrinsically inherited from the ability of 8-oxoguanine to pair with adenine and are closely related to subsequent DNA repair or RNA surveillance. As a coordinated action for regulatory modification, 8-oxoguanine seems to follow unusual writer, reader, and eraser effectors. ROS directly writes 8-oxoguanine, DNA repair proteins read and/or erase 8-oxo-dG, and RBPs read o^8^G, although the nature of 8-oxoguanine as a result of oxidative damage makes it difficult to decisively categorize it. Furthermore, direct repair of o^8^G is still unknown, but it could present as in the case of an RNA repair system that directly seals ribotoxin-induced breakage of tRNA^204,205^.

Since 8-oxo-dG behaves as a potent oncogenic mutagen (G > T and T > G), its excessive occurrence in the genome is prohibited by a specific BER with the aid of other alternative DNA repair pathways (e.g., MUTYH and MTH1)^32^. However, the remaining 8-oxo-dG can induce transcriptional mutations (C > A)^69^ and deteriorate the integrity of regulatory sequences. 8-Oxo-dG and the subsequent repair intermediate AP site can control transcription by interacting with repair proteins (OGG1 and APE1), which recruit transcriptional regulators for functional elements (e.g., G-quadruplex and NF-kB binding sites)^14,15^. In addition to interfering with CpG methylation (DNMT1 and MBP), 8-oxo-dG also mediates transcriptional regulation in association with other epigenetic modifications (histone and CpG island methylation), where local ROS production, generated by the demethylation process (LSD1 and TET1), modifies resident elements to 8-oxo-dG. The recent development of 8-oxo-dG sequencing has now set the stage for the study of its distribution in the genome^112^. Future investigation should aim to advance the precision of 8-oxo-dG genome sequencing to yield proper insights into the epigenetic roles of 8-oxo-dG, particularly in conjunction with other transcriptional regulators and epigenetic modifications.

However, little is known about o^8^G, despite its prominent quantity (more than that of oxidized DNA) in ROS-related diseases^126^. Incorporation of o^8^GTP during transcription can induce a translational error^137^, and o^8^G in mRNA typically degenerates its coding capacity, stalls the ribosome, and produces abortive peptides^133,135^, which are then subjected to NGD and ribosome quality control, leading to selective mRNA degradation^12,123^. Regardless of ribosomes, several o^8^G-bound ribonucleases (PNPase and APE1) and RBPs (YB-1 and AUF1) facilitate o^8^G-RNA decay^153,206^. While o^8^G-mediated translational repression and RNA degradation appear to protect against oxidative RNA damage, these regulations can be interpreted as epitranscriptional regulations for global and selective downregulation of gene expression in response to the redox state. In line with this, some o^8^G-bound RBPs regulate apoptotic signaling pathways (PCBP1 and PCBP2), as o^8^GTP modulates small G proteins in signaling cascades^5,16^. In general, RNA oxidation triggers the repression of global translation through o^8^G modification in rRNA and tRNA, accompanied by their destabilization^16^. However, o^8^G in regulatory noncoding RNAs such as miRNAs (e.g., miR-1) can reprogram their regulatory targets and function via o^8^G•A base pairing^193,194^. Such position-specific o^8^G is likely to be used in noncoding RNAs, including rRNA and tRNA, where o^8^G is likely involved in oxidative stress-induced cleavage to produce their regulatory forms.

Based on o^8^G immunoprecipitation, o^8^G-containing RNAs have been identified using microarrays in an ALS mouse model^134^ or high-throughput sequencing in H_2_O_2_-treated yeast^207^ and bacteria^197^ and in air pollution-^208^ or formaldehyde-treated^209^ bronchial epithelial cells. Nevertheless, signal-to-noise issues have been raised for the mild immunoprecipitation conditions these studies used; thus, these techniques were recently revised for sequencing of o^8^G in cardiac miRNAs and confirmed to outperform with single-nucleotide resolution^193^ relative to the previous conditions^192^. Further studies should be conducted to improve the application of the o^8^G sequencing method to various redox-related diseases. By determining the transcriptome-wide distribution of o^8^G, our knowledge of o^8^G can be expanded, particularly to investigate whether o^8^G interacts with other RNA modifications. The biological significance of 8-oxoguanine is now expanding to its regulatory role in redox-mediated epigenetic and epitranscriptional modifications. However, many aspects of the biological functions postulated here need to be confirmed.
